# Risk factors for retinal microvascular impairment in type 2 diabetic patients without diabetic retinopathy

**DOI:** 10.1371/journal.pone.0202103

**Published:** 2018-08-09

**Authors:** Dong-Hyun Lee, Ho Chul Yi, So Hyun Bae, Joon Hee Cho, Sang Wook Choi, Hakyoung Kim

**Affiliations:** 1 Department of Laboratory Medicine, Gyeongsang National University Hospital, Jinju, Korea; 2 Department of Ophthalmology, Kangnam Sacred Heart Hospital, Hallym University, Seoul, Korea; 3 Hyemin Eye Hospital, Seoul, Korea; Weill Cornell Medicine-Qatar, QATAR

## Abstract

**Objectives:**

To determine the risk factors for retinal microvascular impairment on optical coherence tomography angiography (OCT-A) in type 2 diabetic patients without clinical diabetic retinopathy (DR).

**Methods:**

This retrospective and cross-sectional study enrolled 74 diabetic patients without clinically evident DR for the study group and 34 healthy subjects for the control group. OCT-A parameters were measured to determine vascular density (VD) and the foveal avascular zone (FAZ) size in the superficial and deep capillary plexuses (SCP/DCP) of the retina. Clinical data were collected on sex, age, diabetes duration, hemoglobin A1c (HbA1c), hypertension, dyslipidemia, low-density lipoprotein cholesterol (LDL-C), estimated glomerular filtration rate (eGFR) and smoking status. Multiple linear regression analyses were performed to represent the associated clinical variables with OCT-A parameters in diabetic patients.

**Results:**

In comparison between the study and control groups, the VD in the SCP and DCP were significantly lower in diabetic patients compared to the controls (*P* = 0.022 and 0.003, respectively). The FAZ size in the SCP and DCP were significantly greater in diabetic patients compared to the controls (*P* = 0.035 and <0.001, respectively). In age- and sex-adjusted multiple regression analyses for the diabetic patients, dyslipidemia and hypertension were negatively associated with SCP-VD (β = -0.357, *P* = 0.002; β = -0.239, *P* = 0.039, respectively). Current smoking was correlated with lower DCP-VD (β = -0.255, *P* = 0.043). Greater SCP-FAZ size was associated with dyslipidemia and greater LDL-C (β = 0.254, *P* = 0.013; β = 0.232, *P* = 0.029, respectively), and greater DCP-FAZ size, with lower eGFR and greater LDL-C (β = -0.355, *P* = 0.004; β = 0.235, *P* = 0.037, respectively).

**Conclusions:**

Diabetic patients without clinical DR showed lower VD and greater FAZ size in the SCP and DCP compared to healthy controls. In diabetic patients without clinical DR, dyslipidemia and/or high LDL-C were important risk factors for retinal microvascular impairment. Hypertension, current smoking and lower eGFR also contributed to microvascular impairment.

## Introduction

Many imaging modalities including fundus photography, fluorescein angiography (FA), and optical coherence tomography (OCT) have been widely employed for screening and monitoring of diabetic retinopathy (DR). For the past several decades, FA, providing detailed information on early vascular changes, has been the gold standard for retinal angiography. However, due to the invasive nature of FA, FA-assisted determination of the early structural changes of the retinal microvasculature prior to clinical DR onset has not been widely conducted in general clinical practice. OCT angiography (OCT-A), the most recently introduced of the relevant imaging modalities, enables dye-less in vivo depth-resolved visualization of the retinal microvasculature, which, earlier, was visible only by histologic examination. It is well known that the retinal microvasculature consists of a multilayered complex of capillaries [1]. OCT-A allows for separate visualization of those superimposed retinal capillary layers, whereas the FA cannot image the deeper capillary layer. Additionally, OCT-A, relative to FA, enables more accurate and higher-resolution quantitative analysis of the retinal capillary network. In fact, with the advent of OCT-A, research already has described, in diabetic patients without clinically evident DR, several preclinical changes on OCT-A images, including remodeling of the foveal avascular zone (FAZ), capillary nonperfusion, and reduced capillary density [2–6].

To date, many clinical trials [7–15] have reported numerous risk factors for onset and progression of DR, including duration of diabetes [7], poor glucose [8,9] and blood pressure (BP) control [10–12], diabetic nephropathy [13,14] and dyslipidemia [15], all according to the Early Treatment Diabetic Retinopathy Study (ETDRS) criteria [16], the current gold standard for grading of DR. Recently, an OCT-A-based study reported, for diabetic patients with various severities of DR, several systemic factors related to retinal microvascular impairment including hyperlipidemia, smoking, and renal impairment [17]. Risk factors affecting retinal microvascular changes prior to clinical DR onset, however, have yet to be clarified. In this study, we aimed to investigate the risk factors affecting retinal microvascular impairment as assessed by OCT-A in type 2 diabetic patients showing no clinical evidence of DR.

## Materials and methods

This study was a retrospective, cross-sectional study based in part on our previous report on a pilot study analyzing the relationship between OCT-A features and several risk factors [18]. It was approved by the Institutional Review Board of Kangnam Sacred Heart Hospital, Hallym University (No. 20170215) and the IRB waived the requirement for informed consent. This study was conducted in accordance with the tenets of the Declaration of Helsinki.

### Participants

We retrospectively reviewed the medical records of type 2 diabetes (T2DM) patients who had been monitored by physicians and screened for DR by one retinal specialist (SHB) at Kangnam Sacred Heart Hospital from January 1, 2016 to February 1, 2017. In addition, we reviewed the medical records of healthy subjects who had complained of unilateral symptomatic vitreous floater during the same period. Normal contralateral eyes of healthy subjects served as the control group. T2DM had been diagnosed according to the American Diabetes Association criteria [19]. Our cohort included patients with no clinical evidence of DR (based on the ETDRS criteria) who had been examined using OCT-A. The exclusion criteria of both study and control groups were as follows: < 25 or > 80 years of age, type 1 diabetes, pregnancy, any history or clinical evidence of chorioretinal diseases such as age-related macular degeneration, retinal vascular disease, abnormal vitreoretinal interface, optic nerve diseases such as uncontrolled glaucoma, high myopia of spherical equivalent greater than -7.0 diopters or axial length greater than 26 mm, a history of any intraocular surgery except uncomplicated cataract surgery, and poor-quality images of OCT-A with signal strength index lower than 50. If both eyes of the same patient met the inclusion criteria, one eye was chosen randomly.

### Ophthalmologic examinations

All of the subjects in the study and control groups underwent comprehensive ophthalmologic examinations including slit-lamp biomicroscopy, dilated fundus examination and measurement of intraocular pressure and refractive error. Axial length was measured using Lenstar LS 900 biometer (Haag-Streit AG, Koeniz, Switzerland). Cross-sectional OCT images of the retina were obtained in order to rule out the presence of any vitreoretinal abnormalities. Color fundus photography was acquired for each patient.

We obtained OCT-A images by swept-source OCT (SS-OCT; DRI OCT Triton, Topcon Corporation, Tokyo, Japan), which utilizes a central wavelength of 1050 nm with an A-scan rate of 100,000 scans per second and in-depth digital resolution of 2.6 μm. The OCT intensity information is analyzed using a ratio method, namely OCTA Ratio Analysis, by which the full spectrum is maintained intact and the axial resolution is preserved [20]. A 3 mm x 3 mm square OCT-A image, centered on the fovea, was acquired at a resolution of 320 x 320 in each patient. This OCT-A device provides the en face images of the superficial and deep capillary plexuses (SCP and DCP) which are automatically segmented by the embedded OCT-A software (ImageNet V.6). We manually modified the segmentation in depth of retina in order to reduce the segmentation error if needed.

**Fig 1:**
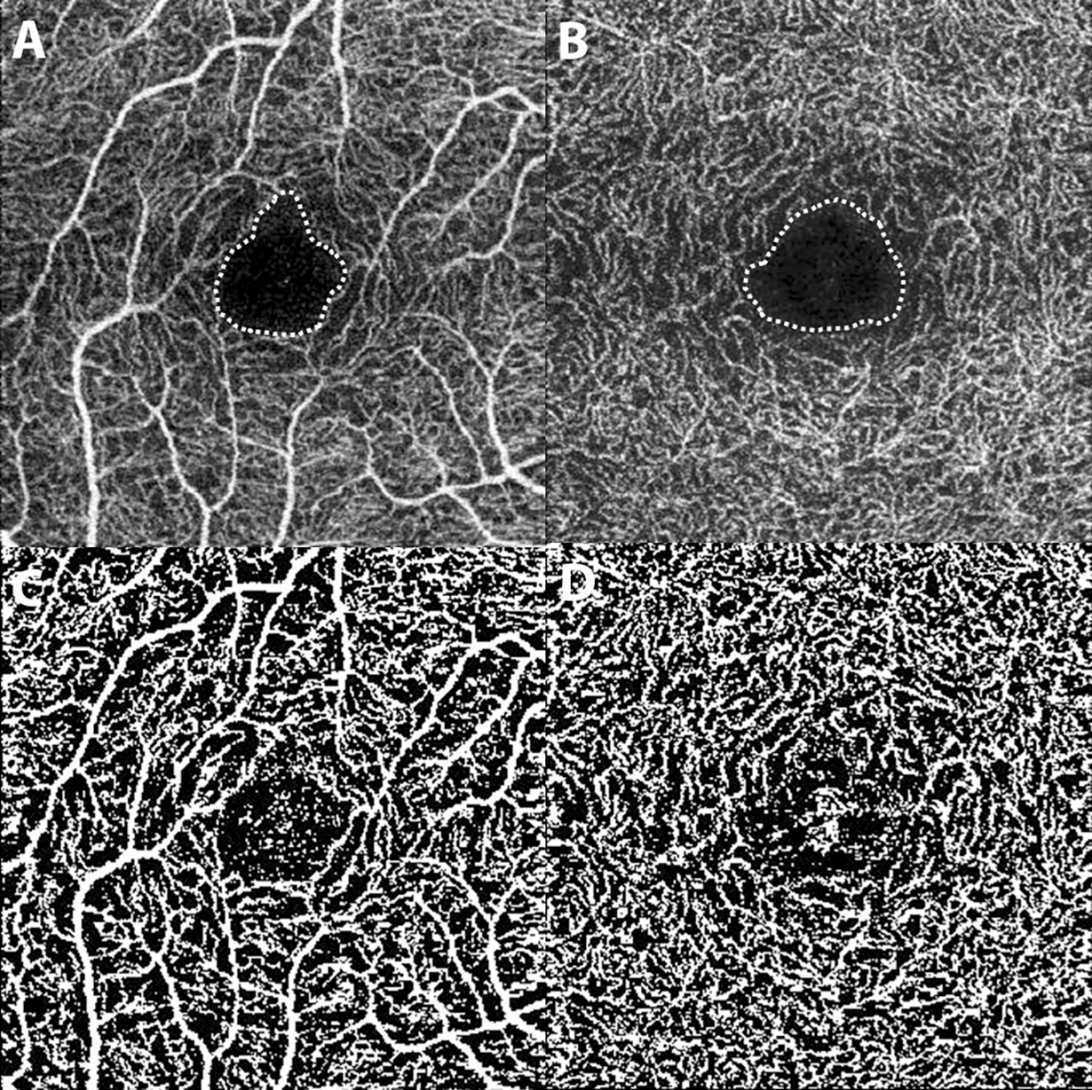
Retinal microvasculature assessed by optical coherence tomography angiography (OCT-A). The foveal avascular zone is outlined manually with a dotted line in superficial (A) and deep capillary plexuses (B). OCT-A images binarized by ImageJ using Niblack method for measurement of vascular density in superficial (C) and deep capillary plexuses (D).

We measured the vascular density (VD) and FAZ size in both the SCP and DCP in order to represent the retinal microvascular status. VD was defined as the ratio of the area occupied by the vessel lumens. To calculate the VD, en-face OCT-A images were analyzed by ImageJ software version 1.5 (National Institutes of Health, Bethesda, Maryland, USA) using the modified Niblack thresholding technique [17,21]. The image was converted to 8 bits and binarized by Niblack Auto Local Threshold method with the mean pixel value with standard deviation (SD) for all the points. The luminal area was highlighted with the brightness set to 0 and 254 [17]. The VD was measured as the automatically calculated ratio of light pixels representing the vessels to the entire pixels of scanned area (3 x 3 mm) using the Threshold Tool. To measure the FAZ size, the border of the retina’s central capillary-free zone was delineated manually using the built in OCT-A software. Then, the embedded software automatically calculated the total area within the outlined FAZ and provided the values in μm^2^. A representative case is shown in Fig 1.

Two trained graders (Yi HC, Choi SW) masked to all clinical information independently reviewed the fundus photographs and OCT-A images. In three cases with disagreement of FAZ size, a consensus was derived to reduce the differences less than 10% of measured values. Then, the average measurements were used for analysis.

### Medical history and biochemical markers

Clinical data were collected based on physicians’ charts and patients’ self-reports. We obtained information on age, sex, duration of diabetes, presence of hypertension (HTN), dyslipidemia and chronic kidney disease (CKD) as well as a documented history on any coronary arterial diseases and cerebrovascular disease, types of DM treatment, and use of antihypertensive, anti-platelet or lipid-lowering medications. Coronary artery disease included a history of angina, myocardial infarction or coronary revascularization such as coronary artery bypass surgery or angioplasty. Cerebrovascular disease included a history of stroke or revascularization at the carotid arteries. HTN was defined as BP of 140/90 mmHg or more or anti-hypertensive treatment. Dyslipidemia was defined as total cholesterol ≥ 200 mg/㎗, triglycerides (TG) ≥ 150 mg/㎗, low-density lipoprotein cholesterol (LDL-C) ≥ 130 mg/㎗ or high-density lipoprotein cholesterol (HDL-C) < 40 mg/㎗ or use of lipid-lowering medication [22]. The stages of CKD were assigned based on the Kidney Disease: Improving Global Outcomes (KDIGO) classification [23] as follows (estimated glomerular filtration rate (eGFR) in ml/min/1.73m^2^): stage 1 (eGFR≥90); 2 (eGFR <90 and ≥60); 3 (eGFR <60 and ≥30); 4 (eGFR <30 and ≥15); and 5 (eGFR <15). Subsequently, those stages were group-categorized as follows: no CKD, stage 1–2 CKD, or ≥ stage 3 CKD. Body mass index (BMI) was calculated as weight in kilograms divided by the square of the patient’s height in meters.

The laboratory data were reviewed within a time window of 3 months from acquisition of OCT-A images. After overnight fasting, blood samples were taken. Total plasma cholesterol, TG and HDL-C was measured using the Hitachi 7600 analyzer (Hitachi, Ltd., Tokyo, Japan), and LDL-C was calculated using the Friedewald formula [24]. Glycated hemoglobin (HbA_1c_) was determined by high-performance liquid chromatography (HPLC; D-100 analyzer, Bio-Rad, Hemel-Hempstead, UK). The value of eGFR was calculated using the equation utilized in the Modification of Diet in Renal Disease (MDRD) study [25].

### Statistical analysis

Continuous variables are presented as the mean (SD) or median (IQR, interquartile range) according to the normality distribution by Kolmogorov-Smirnov test and Q-Q plot. Categorical variables are given as numbers or percentages. The variables of diabetes duration, HbA_1c_ and BMI were skewed distributed, after which HbA_1c_ and BMI were natural log-transformed prior to analyses. However, this study, by design, included a relatively high ratio of newly-diagnosed T2DM patients (19 cases, 26%); thus, the data on diabetes duration were significantly right-skewed, resulting in failure of transformation to the normal distribution.

The comparisons between the study and control groups were conducted using chi square test or independent *t* test. In the study group, univariate linear regression analyses were performed on all of the clinical variables and OCT-A parameters including VD and FAZ area in both the SCP and DCP. The examined clinical variables were as follows: sex, age, age at diabetes diagnosis, diabetes duration, Ln (BMI), former/current smoker, Ln (HbA_1c_), presence of HTN or dyslipidemia, systolic and diastolic BP, total cholesterol, TG, LDL-C, HDL-C, hemoglobin, eGFR, stages of CKD, types of DM medication, and use of antihypertensive, anti-platelet or lipid-lowering medication (statins and/or fibrates). Variables associated with OCT-A parameters with a P-value less than 0.1 were entered into the multiple linear analyses based on stepwise elimination. The variance inflation factor (VIF) was used to detect collinearity among the independent variables. If the calculated VIF was 3.0 or greater, the variable was removed from the model. Statistical analyses were performed using SPSS software version 24.0 (IBM-SPSS, Chicago, Illinois, USA). The results with P < 0.05 were considered statistically significant.

## Results

A total of 74 Korean patients with T2DM for the study group and 34 healthy subjects for the control group were enrolled in this study. The mean age in the study group was 56.6 ± 12.8 years and 60.1 ± 13.1years in the control group. Forty-four patients (60%) of the study group and 16 (47.1%) of the control group were male. There were no significant differences in age and sex between the study and control groups (P = 0.19 and 0.228, respectively).

The mean VD measured by OCT-A in the SCP and DCP for the study group were 35.3 ± 0.88% (range, 32.84 to 37.66%) and 34.8 ± 0.79% (range, 32.82 to 36.48%), respectively, while those for the control group were 35.74 ± 0.93% (range, 33.87 to 37.49%) and 35.33 ± 0.86% (range, 33.34 to 36.73%), respectively. The VD in the SCP and DCP were significantly lower in the diabetic patients compared to normal controls (P = 0.022 and 0.003, respectively). The mean FAZ size in the SCP and DCP for the study group were 0.38 ± 0.13 mm^2^ (range, 0.13 to 0.74 mm^2^) and 0.67 ± 0.20 mm^2^ (range, 0.26 to 1.14 mm^2^), respectively, while those for the control group were 0.32 ± 0.09 mm^2^ (range, 0.18 to 0.46 mm^2^) and 0.46 ± 0.1 mm^2^ (range, 0.27 to 0.67 mm^2^), respectively. The FAZ size in the SCP and DCP were significantly greater in the diabetic patients compared to the normal controls (P = 0.035 and <0.001, respectively).

The clinical characteristics of the study group are summarized in Table 1. Their median (IQR) duration of diabetes was 3.1 (0, 10.3) years. Median (IQR) HbA_1c_ and BMI were 8.0% (6.7, 10.2) and 25.6 kg/m^2^ (23.3, 27.9), respectively. Nineteen (19; 26%) of the patients were current smokers. The prevalences of HTN and dyslipidemia were 54% (40 patients) and 73% (54 patients). The prevalence of coronary artery and cerebrovascular disease were 15% (11 patients) and 11% (8 patients). Seven patients (10%) had CKD stages 3–5.

**Table 1:** Clinical characteristics of diabetic patients with no evidence of diabetic retinopathy.

	Participants
Number of patients, n	74
Male, n (%)	44 (60)
Age, years (SD)	56.6 (12.8)
Age at diabetes diagnosis, years (SD)	50.4 (12.6)
Diabetes duration, years [IQR]	3.1 [0, 10.3]
Body mass index, kg/m^2^ [IQR]	25.6 [23.3, 27.9]
Smoking, n (%)	
‧ Former	17 (26)
‧ Current	19 (26)
Hemoglobin A1c, % [IQR]	8.0 [6.7, 10.2]
Hypertension, n (%)	40 (54)
Dyslipidemia, n (%)	54 (73)
Systolic blood pressure, mmHg (SD)	121.0 (15.1)
Diastolic blood pressure, mmHg (SD)	75.3 (8.6)
Total cholesterol, mg/㎗ (SD)	185.7 (39.0)
Triglycerides, mg/㎗ (SD)	166.3 (99.9)
Low-density lipoprotein cholesterol, mg/㎗ (SD)	109.4 (31.2)
High-density lipoprotein cholesterol, mg/㎗ (SD)	47.4 (10.7)
Hemoglobin, g/㎗ (SD)	14.1 (1.7)
eGFR, ㎖/min/1.73m^2^ (SD)	89.9 (23.1)
Chronic kidney disease, n (%)	
‧ Stages 1–2	34 (46)
‧ Stage ≥ 3	7 (10)
Any coronary artery disease, n (%)	11 (15)
Any cerebrovascular disease, n (%)	8 (11)
Diabetes treatment, n (%)	
‧ Oral hypoglycemic agent	41 (55)
‧ Insulin	13 (18)
‧ Insulin and oral hypoglycemic agent	20 (27)
Use of anti-hypertensive medication, n (%)	39 (53)
Use of anti-coagulant medication, n (%)	27 (37)
Use of lipid-lowering medication, n (%)	39 (53)
‧ Statins	37 (50)
‧ Fibrates	4 (5)

eGFR = estimated glomerular filtration rate.

Data are presented as mean (SD, standard deviation), median [IQR, interquartile range] or number (percentages) as appropriate.

The univariate linear regression analysis results for the clinical variables versus each OCT-A parameter in the study group are shown in Table 2. Lower VD in the SCP was significantly associated with greater diabetes duration, HTN and dyslipidemia (P = 0.026, 0.001 and <0.001, respectively). The variables associated with lower VD in the DCP were older age, lower Ln (BMI) and current smoking (P = 0.047, 0.03 and 0.02, respectively). The greater FAZ size in the SCP was significantly associated with the following variables: female sex, dyslipidemia, greater total cholesterol, and greater LDL-C (P < 0.001, 0.007, 0.011, and 0.02, respectively). The variables correlated with greater FAZ size in the DCP were older age at diabetes diagnosis, dyslipidemia and lower eGFR (P = 0.033, 0.046 and 0.003, respectively).

**Table 2:** Univariate analysis of risk factors associated with retinal microvascular impairment assessed by optical coherence tomography angiography in diabetic patients.

Risk factors	Capillary vascular density, %				Area of foveal avascular zone, mm^2^			
	Superficial capillary plexus		Deep capillary plexus		Superficial capillary plexus		Deep capillary plexus	
	β^a^	P-value	β^a^	P-value	β^a^	P-value	β^a^	P-value
Male	-0.126	0.285	0.026	0.827	-0.431	<0.001^d^	-0.228	0.051
Age, years	-0.193	0.099	-0.232	0.047^d^	0.065	0.58	0.17	0.148
Age at diabetes diagnosis, years	-0.056	0.635	-0.172	0.143	0.082	0.486	0.248	0.033^d^
Diabetes duration, years	-0.258	0.026^d^	-0.113	0.336	-0.028	0.813	-0.138	0.241
Ln (BMI), kg/m^2^	0.036	0.763	0.269	0.03^d^	-0.062	0.599	-0.161	0.17
Former smoker^b^	-0.076	0.522	-0.203	0.082	-0.159	0.175	-0.074	0.53
Current smoker^b^	-0.092	0.437	-0.271	0.02^d^	0.117	0.322	0.009	0.942
Ln (HbA1c), %	0.039	0.741	-0.077	0.516	-0.104	0.377	0.134	0.254
Hypertension	-0.395	0.001^d^	-0.008	0.946	0.019	0.875	-0.016	0.892
Dyslipidemia	-0.424	<0.001^d^	0.021	0.856	0.31	0.007^d^	0.233	0.046^d^
Systolic blood pressure, mmHg	-0.13	0.27	0.088	0.458	0.003	0.981	-0.001	0.996
Diastolic blood pressure, mmHg	0.134	0.256	0.172	0.142	0.043	0.718	-0.03	0.799
Total cholesterol, mg/㎗	-0.012	0.917	0.169	0.15	0.294	0.011^d^	0.145	0.217
Triglycerides, mg/㎗	-0.043	0.718	0.181	0.123	-0.024	0.841	-0.066	0.578
LDL-C, mg/㎗	0.08	0.496	0.129	0.273	0.269	0.02^d^	0.201	0.087
HDL-C, mg/㎗	0.067	0.573	-0.122	0.299	0.225	0.054	0.151	0.199
Hemoglobin, g/㎗	0.109	0.356	0.076	0.518	-0.068	0.566	0.13	0.27
eGFR, ml/min/1.73m^2^	0.089	0.45	0.1	0.395	-0.114	0.335	-0.338	0.003^d^
Chronic kidney disease^c^								
‧ Stages 1–2	-0.087	0.466	0.167	0.157	-0.181	0.125	0.023	0.845
‧ Stage ≥ 3	-0.048	0.687	-0.138	0.243	0.095	0.424	0.065	0.585
Coronary artery disease	-0.097	0.41	0.065	0.585	-0.047	0.694	-0.203	0.083
Cerebrovascular disease	-0.105	0.373	-0.014	0.906	0.071	0.546	0.041	0.727

eGFR = estimated glomerular filtration rate; BMI = body mass index; HbA_1c_ = glycated hemoglobin; HDL-C = high-density lipoprotein cholesterol; LDL-C = low-density lipoprotein cholesterol.

^a^Indicates standardized coefficient.

Reference categories are the groups with

^b^non-smokers

^c^without chronic kidney disease.

^d^P-value < 0.05

The multiple linear regression analysis results after controlling for age and sex in the study group are presented in Table 3. Lower VD in the SCP was significantly and negatively correlated with dyslipidemia (standardized coefficient [β] = -0.357; 95% CI, -0.573 to -0.141; P = 0.002) and HTN (β = -0.239; 95% CI, -0.466 to -0.012; P = 0.039). As for lower VD in the DCP, current smoking was the only associated factor (β = -0.255; 95% CI, -0.502 to -0.008; P = 0.043). Greater FAZ size in the SCP was significantly associated with dyslipidemia (β = 0.254; 95% CI, 0.054 to 0.45; P = 0.013) and increased LDL-C (β = 0.232; 95% CI, 0 to 0.464; P = 0.029). Greater FAZ size in the DCP, meanwhile, was associated with decreased eGFR (β = -0.355; 95% CI, -0.592 to -0.118; P = 0.004) and increased LDL-C (β = 0.235; 95% CI, 0 to 0.353; P = 0.037).

**Table 3:** Multivariate analysis of age- and sex-adjusted risk factors associated with retinal microvascular impairment assessed by optical coherence tomography angiography in diabetic patients.

	β^a^, [95% CI]	P-value	Adjusted R^2^
Capillary vascular density in SCP, %			
‧ Age, years	-0.08, [-0.444, -0.043]	0.47	0.23
‧ Male	-0.108, [-0.318, 0.102]	0.309	
‧ Dyslipidemia	-0.357, [-0.573, -0.141]	0.002	
‧ Hypertension	-0.239, [-0.466, -0.012]	0.039	
Capillary vascular density in DCP, %			
‧ Age, years	-0.189, [-0.41, 0.047]	0.113	0.074
‧ Male	-0.034, [-0.285, 0.217]	0.787	
‧ Current smoking	-0.255, [-0.502, -0.008]	0.043	
Area of foveal avascular zone in SCP, mm^2^			
‧ Age, years	0.187 [0.0, 0.374]	0.076	0.289
‧ Male	-0.405, [-0.604, -0.203]	<0.001	
‧ Dyslipidemia	0.254, [0.054, 0.45]	0.013	
‧ LDL-C, mg/㎗	0.232, [0.0, 0.464]	0.029	
Area of foveal avascular zone in DCP, mm^2^			
‧ Age, years	0.106 [-0.106, 0.318]	0.189	0.199
‧ Male	-0.255 [-0.47, -0.042]	0.02	
‧ eGFR, ml/min/1.73m^2^	-0.355, [-0.592, -0.118]	0.004	
‧ LDL-C, mg/㎗	0.235, [0.0, 0.353]	0.037	

CI = confidence interval; DCP = deep capillary plexus; eGFR = estimated glomerular filtration rate; LDL-C = low-density lipoprotein cholesterol; SCP = superficial capillary plexus.

^a^ Indicates standardized coefficient.

## Discussion

Our study showed that diabetic patients without clinical DR have significantly lower VD and greater FAZ size in both SCP and DCP compared to normal controls in accordance to previous reports [2–6]. The cross-sectional studies done by Dimitrova et al [3] and Cao et al [6] have documented the significant reduction in superficial and deep VD in diabetic patients without DR compared to the control groups consistent with our results. They suggested that the compromised retinal microcirculation would occur prior to clinical manifestation of DR [3,6]. In addition, many studies reported significantly greater FAZ size in diabetic patients compared to the healthy subjects consistent with our results [2–5]. Whereas Cao et al [6] and Goudot et al [26] did not find significant differences in FAZ size between diabetic patients and normal controls. Cao et al [6] have suggested this discrepancy might be caused by wide variation of the FAZ size in healthy individuals. However, herein, we speculated that the greater FAZ size also could be a marker for early retinal microvascular changes in diabetic patients prior to development of DR based on our findings.

In the current study, we also evaluated the systemic risk factors for retinal microvascular impairment as assessed by OCT-A in T2DM patients with no clinical evidence of DR. Lower VD in the SCP was independently associated with dyslipidemia and HTN, whereas, in the DCP, current smoking was the only associated factor for lower VD. We also found that dyslipidemia and greater LDL-C were significant factors determining greater FAZ size in the SCP, whereas in the DCP, lower eGFR and greater LDL-C were.

Considered together, our results suggest that dyslipidemia and/or high LDL-C is one of the major risk factors for early retinal microvascular impairment prior to onset of clinically apparent DR. Previously, clinical trials have presented conflicting results on the contribution of lipid abnormalities to onset or progression of DR [15,27–32]. The Wisconsin Epidemiologic Study of Diabetic Retinopathy (WESDR) reported that total cholesterol was not a significant factor in DR severity [28]. In the Diabetes and Complications Trial/Epidemiology of Diabetes Interventions and Complications Study (DCCT/EDIC), however, DR severity was positively associated with TG and negatively associated with HDL-C [15]. Also, the presence of retinal hard exudate or macular edema has been correlated with lipid abnormalities such as elevated total cholesterol [28,29,31], LDL-C [29–31] or TG [31]. Recently, an OCT-A study showed that low capillary VD in T2DM patients was associated with hyperlipidemia [17], which finding supports our results. It has been suggested that several hyperglycemia-triggered metabolic pathways such as protein kinase C activation and advanced glycation end-product formation interact with lipids, thereby leading to abnormalities in the retinal capillary bed [33,34].

Additional risk factors found in multivariate analyses to be associated with retinal microvascular impairment on OCT-A are the presence of HTN, lower eGFR and current smoking. Epidemiological studies have identified that HTN increased the risk of development and progression of DR [10–12]. Kohner [35] suggested that hyperglycemia impaired autoregulation of retinal microvasculature and made the retinal microvascular endothelium prone to damage by high blood pressure. In addition, the experimental study done by Suzuma et al proposed that HTN-induced mechanical stress on the retinal endothelium might contribute to deterioration of DR, specifically via increased expression of vascular endothelial growth factor (VEGF) receptors [36]. Our results also demonstrated that HTN was one of risk factors for retinal microvascular impairment measured by OCT-A, although its effect was limited to only VD in the SCP. Abnormal renal profiles including albuminuria [13,14] and low eGFR [37,38] also have been reported as risk factors for progression of DR. Hsieh et al has suggested that the production of VEGF secondary to glomerular injury in CKD might increase the VEGF level in systemic circulation, resulting in progression of DR [37]. The effect of smoking in the progression of DR is controversial. The United Kingdom Prospective Diabetes Study (UKPDS) group demonstrated that current smokers had a reduced progression of DR compared with never smokers [39]. However, recent studies using blood flowmeter [40] or OCT-A [17] reported that smoking was related with reduced retinal blood or low VD in diabetic patients, compatible with our results.

There is an established causal relationship between DR and degree or duration of hyperglycemia [7–9]. However, our results did not indicate any relationship between the OCT-A-measured parameters and HbA_1c_ or duration of diabetes, other than a negative association of the latter with SCP-VD in the univariate linear regression analysis. In this study, we assessed diabetic patients without clinically evident DR, which selection contributed to the relatively short duration of diabetes (median: 3.1 years) and the high ratio of newly-diagnosed diabetic patients (26%) among our cohort. Among the DCCT cohort, total glycemic exposure, compounded by the multiplicative effect of the level of HbA_1c_ with the duration of exposure to hyperglycemia, was the dominant factor relevant to DR progression [41]. Thus, we think that the range of total glycemic exposure in our study might be insufficient to make possible any statistically significant difference in retinal microvascular impairment among the enrolled patients.

In our results, the risk factors were variable according to the different capillary layers. The SCP and DCP’s different features on OCT-A [42,43] might have affected those results. The DCP is composed of polygonal units converging radially into epicenters [42], which drain into the superficial venules through vertical anastomoses [42,43], whereas the SCP is constituted of transverse capillaries directly connected to the retinal arterioles with higher perfusion pressure [42]. Based on these anatomies, it has been suggested that the DCP might be more vulnerable to ischemia than is the SCP [44,45]. In any case, our present conclusions and suppositions require further supporting evidence related to the hemodynamics of each retinal microvascular network.

The strengths of this study include multivariate-analysis-based assessment of a wide range of systemic risk factors possibly affecting retinal microvascular changes. Whereas an earlier OCT-A study investigated several risk factors for diabetic patients manifesting various severities of DR [17], we limited our focus to diabetic patients showing no signs of clinical DR. This approach could be the first step to developing a noninvasive predictive model correlated with preclinical retinal microvascular impairment in diabetic patients. However, our study also has limitations such as its cross-sectional nature and relatively small sample size. Further, large-sample longitudinal studies are warranted for evaluation of the efficacy of risk factors in predicting DR onset and progression. Among the other limitations of the present study, the OCT-A scanning window was limited to the central macula, which does not represent the entire retina. Also, OCT-A image artifacts can hinder proper assessment of the actual status of the retinal microvasculature; for example, the projection artifacts might interrupt the visualization of the deep layer. Another limitation of this study is that we used single OCT-A device. It is known that the measurements from the various OCT-A devices are not interchangeable [46,47]. The currently available OCT-A instruments used different algorithms to quantify the motion contrast and different segmentation boundaries. Several OCT-A devices used automatic software to measure the FAZ size or VD, but it is not available in DRI OCT Triton used herein which might lead to biased results. Thus, further studies using various OCT-A devices are needed to support our results. Furthermore, researchers have measured different OCT-A parameters including FAZ size, VD and fractal dimension, and have used various means of OCT-A image manipulation, all of which variances have the potential to affect results. Lastly, we enrolled the patients who were monitored by physician and retinal specialist in the same hospital to obtain wide range of medical information. In addition, the retinal screening was performed by single retinal specialist (SHB) among three retinal specialists in our clinic. Those might raise the possibility of selection bias.

In conclusion, we demonstrated the significantly lower VD and greater FAZ size measured by OCT-A in T2DM patients without clinical DR compared to healthy controls. This study, having investigated almost all of the OCT-A parameters relevant to T2DM patients showing no evidence of clinical DR, determined that dyslipidemia and/or high LDL-C were the important risk factors for retinal microvascular impairment. Hypertension, current smoking and lower eGFR also contributed to retinal microvascular impairment.

## Supporting information

S1 File The values of OCT-A parameters and clinical characteristics of the study and control groups. (XLSX)
